# O4-1 A scoping study of the international implementation of the World Health Organisation's global physical activity action plan

**DOI:** 10.1093/eurpub/ckac094.025

**Published:** 2022-08-29

**Authors:** Jack Walklett, Diane Crone, Vasiliki Kolovou, Nicola Bolton

**Affiliations:** 1 School of Sport and Health Sciences, Cardiff Metropolitan University, Cardiff, United Kingdom; 2 School of Management, Cardiff Metropolitan University, Cardiff, United Kingdom

**Keywords:** GAPPA, systems, whole-system approach

## Abstract

**Background:**

Published in 2018, the World Health Organisation's Global Action Plan for Physical Activity (GAPPA) sets out 20 actions across 4 objectives (Active Societies, Active Environments, Active People, Active Systems) to reduce global inactivity by 15% by 2030. GAPPA takes a whole-systems approach (WSA), calling for cross-sectoral collaboration to address physical inactivity levels. This study sought to explore implementation to date, highlighting progress made and areas that require further focus.

**Methods:**

A desk-based scoping study, using a systematic, but flexible approach, identified sites and personnel implementing GAPPA or a WSA. Methods of collection included a review of existing literature, snowball sampling of key individuals and internet searches. These methods identified a range of academics and practitioners who were subsequently contacted and interviewed on their use of GAPPA to date.

**Results:**

Preliminary findings suggest that GAPPA's implementation currently varies greatly internationally. Some nations, including Finland and Scotland have embraced a WSA, taking steps to actively map their PA system. Elsewhere, countries such as Ireland and Australia are exploring practical approaches to implementing a WSA. GAPPA was only published in June 2018 and thus, where action is taking place, these countries offer important implementation evidence and opportunities for transferable learning. Regarding GAPPA's objectives, our review concludes that in nations that have embraced GAPPA, the Active Societies and Active Environments objectives are being actioned or have plans in development for action, while progress towards behaviour and system change objectives (Active People and Active Systems) appear less evident.

**Conclusions:**

There is some promising evidence drawn from GAPPA's early implementation, although we conclude that if WHO targets are to be reached, wider adoption is needed. It appears that the nations who have best applied GAPPA, are those who arguably have pre-existing cross-sectoral networks in PA and public health. Therefore, perhaps a crucial first step in nationally implementing GAPPA is the establishment of these networks, laying foundations for collaborative action. Furthermore, the generation and dissemination of research by some of the early adopters is crucial to both reinforce knowledge transfer and guide others towards a whole-systems approach.

